# An enhanced heuristic XoR network coding-based method for high quality video streaming over VANETs

**DOI:** 10.1371/journal.pone.0218647

**Published:** 2019-06-28

**Authors:** Maryam Mosaarab, Behrang Barekatain, Kaamran Raahemifar, Homa Movahednejad

**Affiliations:** 1 Faculty of Computer Engineering, Najafabad Branch, Islamic Azad University, Najafabad, Iran; 2 Big Data Research Center, Najafabad Branch, Islamic Azad University, Najafabad, Iran; 3 Electrical and Computer Engineering, Sultan Qaboos University, Al-Khoud, Sultanate of Oman; 4 Chemical Engineering Department, University of Waterloo, Waterloo, Ontario, Canada; University of Zilina, SLOVAKIA

## Abstract

One of the most important challenges in live video streaming in mobile vehicular networks is the optimal use of broadband and point-to-point packet delay. Recent studies show that the sheer use of frames flow compression methods (such as H.264 or HEVC) and the proper communication overlay, such as Peer-to-Peer (P2P), has no absolute influence on increasing the quality of received video in VANET networks. Therefore, the use of an appropriate data exchange method, such as network coding, seems to be of great importance. Compared to Random Network Coding (RNC), XoR Network Coding (XNC) method has the least computational load for the network which is an important factor in optimal use of limited energy of nodes in a wireless network. The basic problem in XNC is that when a node is supposed to combine several frames and transmit them through an encoded frame, how this combination could be made to enable other nodes of the network to be broadcasted through receiving this packet and how can the available packets in their buffers decode as well as extract the largest number of frames in order to experience a higher video quality. To fulfil this aim, an encoding intelligent method is required which is based on the buffers’ status of neighbours. In the proposed method in this article, the best frame combination is reached through buffers status of neighbours and AHP methods or AHP-TOPSIS methods, and the encoded frames are broadcasted through XNC. Simulation results show that due to the reduction in number of transmitted packets in the network, parameters such as congestion and point-to-point delay are significantly reduced and vehicles experience a higher video quality compared with other similar methods.

## Introduction

Nowadays, lots of people use cars or other transport means which leads to a huge number of accidents and fatalities. Notifying drivers of road conditions, traffic and related issues is critical to security and traffic congestion reduction. In this regard, the Vehicular Ad hoc Network (VANET) is an infrastructure for intelligent transportation system, a subset of Mobile Ad hoc Network (MANET) and is not dependent on the fixed infrastructure. Moreover, its nodes have a high mobility rate. To improve the road security and convenience for passengers, VANET creates a wireless communication (using DSRC (Dedicated short-range communications)) among the mobile vehicles. DSRC is essentially IEEE 802.11a amended for low overhead operation to 802.11p [1]. Vehicles are able to communicate directly with each other, which is called Vehicle-to-Vehicle (V2V), or vehicles can communicate with a communication overlay through the road side unit equipment which is shortened as Road Side Units (RSU) that is called Vehicle-to-Infrastructure (V2I) [1–5].

Recently, among many traffic types, video streaming in vehicular networks has been of great interest. In fact, they create amusing and advertising services. As an example, in a road emergency, broadcasting a live video of the crash scene allows the vehicles to make a more correct decision on how to choose a route or how to help the people involved in the accident. Similarly, videos displaying the traffic are also used as a guide to choose the best route. In addition, broadcasting multimedia content for OBUs (On-Boards Units) in a specific area can be considered as an entertaining service. For example, a local hotel launches video advertisements for vehicles which enter the city, a travel agency announces its activities in a tourist area for passing cars, and highway management companies can broadcast movies for travellers during the long distances [6]. In all of these cases, the live video streaming is considered.

In general, there are two types of streaming: live video streaming and video on demand (VoD). In VoD, in which the video streaming is ready to be delivered, users can view the stream when they download it. An example of VoD is online video shops and YouTube. Users usually start watching the video from the beginning. At a moment, users watch different video segments. In live video streaming, the video is compressed and packaged at the same time. All users can see it when the content is being generated. In the meantime, in live streaming, there is a source which broadcasts the video streaming. For example, suppose someone who is presenting a speech, and at the same time all vehicles have to receive this lecture and this speech is broadcasted at almost the same time. As a result, the point-to-point delay for live streaming is more meaningful. Compared to VoD, live streaming has several advantages, for example, live streaming services can support a sufficient amount of broadband; therefore, users can display the streaming content at any time and at any point. That is why the uploading bandwidth increases when the number of users goes up; thus, live streaming services do not require much storage space [7].

A scenario for broadcasting the video in vehicular networks can be the video content broadcasting using RSUs, which means a vehicle downloads a video through license-free wireless spectrum when it is placed in RSU transmission range [6]. Due to the high mobility of nodes and quick changes of topology in this network, designing an efficient routing protocol, which is able to deliver a packet within the least period of time, can increase the efficiency of the network particularly the live video flow that is delay-sensitive [2,7]. The most important issues in the transmission of live video stream are the reduction of network congestion, efficient use of the broadband, and the reduction in the point-to-point delay [7,8].

In the past, several methods have been proposed for saving power and broadband consumption in distributed networks. For example, to save the broadband, a genetic algorithm for optimizing the routes in a multi cast routing scenario [9], or to control the efficiency and delay of the broadband, Forensic-Scheduling design [10] and also, to avoid the resource shortage in route selection process, fuzzy prediction method [11] are proposed.

What can be inferred from recent studies is that, in order to provide high quality video playback on vehicles, we have to address three important issues including video frame size and dependency, an efficient network infrastructure and an efficient method for exchanging video frames among peer.

As the first issue, video frames consume a large amount of bandwidth due to their large size in byte. In other words, as one second of a video stream includes 24 or 30 frames, we need large bandwidth for transferring these frames to the next hop before their playback time. Video encoding is a suitable solution to address this issue [12–14]. In other words, to meet the user’s needs in cases such as transmission speed, quality, and available resources, video encoding is an important stage. The most well-known standard video encoding is H.264/AVC (Advance Video Coding) which is widely used in digital broadcasting [15,16]. In H.264 standard, various orders of frames can be encoded in a Group-of-Picture (GoP).

**Fig 1:**
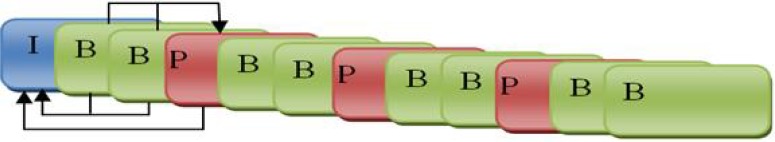
A twelve-frame classic GoP [17].

More precisely, as seen in Fig 1 [17], the GoP structure uses three different types of images, frames I, P, and B. frames I are the reference frames used to retrieve the entire GoP. In other words, all frames based on this frame can be restored and corrected. Frame-I is followed by sequences of frames P and B. Frame-P contains various information from the frames I or P which are placed before them; they are used to retrieve frames P and B before and after them. Retrieving frame-B depends on getting the correct frames P before and after or frames I before. In other words, frame-B is dependent on frames I or P before and after them in a GoP. Frame-I and frame-P are used to compensate for the movement of frames B. Frame-B helps vehicles to provide better video quality for their passengers [15, 18].

Due to the dependence of frames, required frames have to be received by the receiver in the proper time. Otherwise, even the safe reception of them will be of no value. Because for example, B1 frame decoding will not be possible without the previous I and the next P frames. As a result, we need an efficient network infrastructure to reduce the malicious effect of dependency between frames on the quality of the received video.

This infrastructure can be Client/ Server, Content delivery Network (CDN), or Peer-to-Peer (P2P). General technologies of video broadcasting usually use Client/Server model and using CDN to broadcast the media on the Internet. However, these two models are not appropriate, because when a large number of users ask for the content simultaneously, the server requires a high broadband. For addressing this issue, previous studies showed that Peer-to-Peer (P2P) networks, provides efficient communication model for vehicles [18]. In other words, it not only provides an efficient infrastructure among peers for exchanging video frames, but it also decreases the side effect of video frame dependency [19–21].

P2P systems are basically application-level virtual networks with their specific topology and routing methods[22]. P2P Networks provide a scalable, efficient network infrastructure for wireless and wired networks for video streaming [8]. Nodes which participate in P2P Overlay represent their physical network counterpart, but the connection between them is different. Therefore, to make the network more flexible and to increase the variety of the network, P2P Overlay is used. P2P Overlays can be in the form of mesh or tree [19]. Tree-based networks are not resistant enough in peer churning. Moreover, the leaves of the tree do not participate in video streaming that leads to the low efficiency of the network. Not such as tree-based topology, Mesh-based P2P networks create a high throughput in the network and a higher tolerance in peer churning. P2P systems can be structured or unstructured. In structured model, nodes are connected to each other according to a special pattern and there are specific rules concerning the location of peers in topology which are called Distributed Hash Tables (DHTs). Therefore, they are mostly used for file sharing issues in which the location of the data is of great importance. In unstructured model, there is no specific pattern for nodes to be connected (there is no stable topology, and the connections among peers are created randomly) and the node’s membership is free. Theses topologies are more flexible in churning and search operations. Consequently, they are appropriate for real-time traffics such as video streaming [20,23,24].

In other words, vehicles in such a network, with the efficient exchange of packets among them, reduce the delay in frames exchange and cause frames to reach the vehicles in less time. As a result, the effect of the dependency between frames can be reduced. In this regard, vehicles typically form an overlay network on the underlying structure of the VANET network [20, 25].

Although a P2P network creates a good relationship between vehicles, the lack of a proper frame exchange between vehicles can negatively affect all the benefits of these types of networks. This is the third problem. In recent research, methods such as Push, Pull or a combination of them for the frames exchange have been used and all of them have inherent problems due to the two basic methods of Push and Pull. In other words, in the Push method, the high number of duplicate frames in the mesh network and in the Pull method, the delay of the frame exchange is considered to be destructive factors for the live video streaming [17].

The Push method, by sending large duplicate frames, results in the loss of valuable and limited bandwidth between vehicles and increases the delay due to improper use of bandwidth. Pull-based methods also inherently increase the delay, although they reduce the number of duplicated frames. The delay imposed in both of these methods will delay the frames to the vehicles and thus, reduces the quality of the received video. Also, when the delay increases, the effect of the dependency between frames will increase. Although hybrid methods have been proposed to solve this problem, research shows that they are all a simple combination of the two Push and Pull methods, and ultimately lead to disadvantages [26, 27].

As a simple but effective solution, network coding was proposed for non-noise wired networks [25]. Network coding can lead to an efficient broadband consumption, reduction of network collision, and reduction in the number of repeated broadcast transmission. Network coding, by allowing the interfaces to perform some logical operations on packets and sending them, will reduce the number of retransmissions of the network. In network coding, it has been tried to place several packets inside a packet and send several ones instead of sending only one. In vehicular networks, protocols use network coding to transport fewer packets in the network and consume less bandwidth to enhance network stability and reliability. Two main methods for network coding are Random Network Coding (RNC) and XoR Network Coding (XNC) [18,26]. In RNC, a node sends an encoded block which is a random combination of received data by the node [27]. RNC needs no specific knowledge about the network topology. In other words, it is totally topology-independent. Due to the fact that RNC increases the throughput of the network considerably, it has been of great interest for video streaming. However, high transmission overhead, due to sending all coefficient vector entries, can greatly reduce the system performance, especially the live video streaming system. This problem is often seen in wireless networks in which the broadband is limited and mobile vehicles have limited computational resources. Also, RNC involves the CPU a lot. Due to the fact that there are no CPU and powerful process in VANET nodes, XNC is preferred to be used.

Although XNC is a simple method with no specific complexities [23,28–39], the communication structure in vehicular networks has caused XNC to be inefficient, because of communication costs. To improve this method, unnecessary code broadcasting should be reduced. The main challenge for this improvement is to detect unnecessary communication codes [27]. If XNC is performed simply, packets may not be delivered on time and in turn, the video may not have a high quality. In network coding, to enable more vehicles to use XoR combinations in the best way, selecting the best network coding combination is of great importance. Otherwise, vehicles cannot extract their required video frames from the sent encoded frames. This not only leads to low perceived video quality on vehicles, but also the limited bandwidth between two vehicles can be wasted.

In this research, to select the best combination, AHP (Analytic Hierarchy Process) and AHP-TOPSIS (Technique for Order Preference by Similarity to an Ideal Solution) in terms of the conditions of the network traffic) are used. AHP and TOPSIS are among the most well-known Multiple Criteria Decision Making (MCDM) methods which are used to select the best choice with regard to some criteria. AHP has a high dissociation capability and can be used as an appropriate way for choosing the best combination, by proper structuring of the issue and dividing it into simpler parts and weighing the criteria. In addition, it can help TOPSIS which does not represent any special way for weighing the criteria [40].

The use of AHP has been considerably limited due to limited human capacity in information processing. The TOPSIS approach can meet the need for paired comparisons, and capacity constraints do not predominate in the process[41]. As a result, when the number of compounds is made, this number is compared to the threshold. If the number is less than the threshold, then the default AHP method is used. If the number is higher than the threshold, the APH-TOPSIS is used to obtain the best compound. Simulation results show that the proposed method by tis research considerably improved the perceived video quality on vehicles.

The rest of this paper is organized as follows. The following parts contain the related works in the second section and problem statement in the third one. In the fourth section the suggested method is exemplified and in the fifth one, it is simulated. The last section contains the conclusion.

## Related work

This section provides an overview over recent related studies which used network coding for video streaming over VANETs. Based on these studies, we divide this section into two main subsections.

### RNC-based method in vehicular networks

To improve the availability of data in a vehicular network, Linear NC is used. There is an important problem in linear communication among the received codes that reduces the successful performance of data reception rate. Also, the analysis of the effects of a real vehicular environment shows that there is a strong communication structure in a real vehicular network. This communication structure could help the linear dependence issue. Because of the existence of this structure in a real vehicular network, simple network coding for communication costs is inefficient [27]. XNC technique is used for non-safe applications in VANET to decrease the network congestion and to improve the efficiency of the broadband. To make this solution more cost-effective, PRAVDA (Pseudo Random Network Coding in VANET for Data download), a less throughput-based solution which broadcasts through the infrastructure, is used to create lower throughput on each RSU and less collisions with other connections. The main problem with this technique is the high repetition rate which is caused by using various RSUs [42]. To solve this problem, Pseudo RNC has been used to produce a large number of various blocks for reducing overhead which is caused by various RSUs. In the intersection of V2I and V2V communication networks, network coding is used to remove interference in the process of receiving broadcast messages from vehicles still in the broadcast area. As a result, using NC, messages could be sent with the least loss. The quick change in vehicles position can reduce the throughput of the system and increase the error rate of the packet. To solve this, proper Rate-Adaptive methods or power control plans need to be examined more in the future [43]. When several vehicles, which are geographically close to each other, have a common interest in downloading specific stuff, they can cooperate with others to decrease the total download time considerably. The usage of network coding is examined in shared downloading. The probability of information dissemination and the expected amount of time needed to deliver all information to vehicles is obtained with and without NC. Using the network coding can improve the download time. In addition, it eliminates the need for any sort of uplink communication from vehicles to infrastructure [44].

### XOR-based method in vehicular networks

In wireless vehicular networks, mobile nodes move in a high speed. Therefore, the network topology is always changing. When there is a heavy traffic, the collision probability is increased. Network coding is one of the designs for efficient packet-transmission, improving the throughput and reducing the number of packet transmission. The ODT (Opportunistic Delayed Transmission) scheme is proposed to compromise between the delay and coding opportunity where the mid node may be waiting for a certain period of time to maximize the coding opportunity. This scheme not only increases the coding rate, but also the packet-delivery rate [45]. Network coding and Multi-Hop Beaconing have been used in combination to improve the reliability and awareness in vehicular networks. The result shows that Network coding and Multi-Hop Beaconing are able to improve the shared awareness under practical conditions and specific traffic. They can also support the high reliability level of communications in long distances, compared to Single-Hop Beaconing strategies. This combination can also improve the vehicle capacity to communicate with other vehicles in long distances under special conditions. In addition, this combination proves to be useful for solving the problems which are caused by large obstacles such as buildings and trucks[46].

Network coding technique is proposed to improve the utilization of broadband for non-safe application in vehicular networks. In a scenario where there are two sources that transmit data at the same time to the same area, the relay uses a network coding technique to reduce the number of all-retransmission events and optimal bandwidth utilization. Using network coding can lead to an efficient use of broadband, reduction in network congestion and reduction in the number of packet-retransmissions. When using wireless coding in wireless networks, there are random and non-symmetric traffic problems. The design of a transfer policy, which comprises between the delay and broadband usage, is known as ONC (Opportunistic Network Coding). ONC is a scheme which combines traditional routing with network coding and is usually used to compromise between the delay and broadband consumption. When a relay receives a packet, the fundamental problem for it is to decide whether to wait for a coding opportunity and save the broadband, or to send the packet directly and decrease the delay, for which BSCS (buffer size control scheme) and TCS (Time Control Scheme) protocols were introduced to control the delay and optimize the broadband [26].

The BSCS method checks the delay through controlling the buffer size. When the queue length increases, the average delay of each packet will also increase. Therefore, in this method, when the packet arrives, the broadcaster will decide on the queue with respect to the number of the packets available in the queue. In other words, when the packet arrives, the probability of packet queuing is calculated as P = 1/(size of the queue), which is proportional to the queue size. Doing this may cause the new packet to be relayed, while older packets are still waiting in the queue for being encoded and sent. As a result, packets are broadcasted out of order and reach the receiver with a great deal of delay. Thus, instead of queuing the new packet with the probability of P, the broadcaster queues the packet with a 1-P probability and relays it. In TCS, the broadcaster queues all the packets that gradually come from the source. In fact, the broadcaster sets a delay limit for each packet, which results in the fact that the packet cannot be buffered more than a specific time (Tmax). Therefore, when a packet is received from the source, the broadcaster queues it directly, but sets a timer, and after the Tmax, if the packet is still in queue, it will be sent rapidly without encoding, which will increase the bandwidth usage and reduce the performance.

To control the Beacon overhead by packet coding, a mechanism is proposed. This mechanism transmits the network coded Beacons in two steps. In fact, a sending area is selected in transmission range and nodes compete with each other to be selected as the only sender in that area. The proposed protocol is called Network Coding Based Congestion Control (NC-CC), which decreases the Beacon overhead on a channel through the reduction in the transmission rate of the Application- layer in a node. XNC causes the reduction in the number of transmitted Beacons. In turn, the congestion is decreased and a higher broadband is at hand [47]. A scheme was proposed to remove the intervention (due to Beacon communications inside the street) by the transfer control via Multi Hop Beacon Forwarding and XNC for safety applications.

Interventions of other vehicles, which are not part of the shared road segment, are decreased by the reduction in transmission power of each node. The reduction in transmission rate of Beacons lead to the decrease in the channel congestion and using the Packet Level Network Coding, packets can be delivered to receivers in a specific transmission range on time. When the transmitter receives the Beacon messages from two opposite directions, packet level network coding is used to retransmit the result messages and this process continues until the Beacon messages are all sent. The aim is to reduce the transmit power of Beacon messages. As a result, congestion control management and intervention omission are achieved [48].

A new protocol called CNC-MAC (Cooperation and Network Coding based MAC) is proposed for vehicular networks. This protocol utilizes network coding and the cooperative ARQ (Automatic Repeat Request) to increase the throughput through decreasing the transmissions number. This protocol also is more efficient in high mobility and stops the packet loss considerably. Then, the time consumption is decreased significantly [49].

In Table 1, previous related works have been compared.

**Table 1:** A review of related previously-done researches.

Ref.	Research Title	Solution and Method	Difference with the Proposed Method
**[46]**	Congestion Control in Vehicular Networks Using Network coding 2014	Controlling the Beacon overhead using the Packet Level NC	the increase in throughput and the reduction in delay using XOR NC intelligently
**[26]**	Network coding techniques for VANET advertising applications 2015	Controlling the network congestion or delay reduction via BSCS and TCS protocols (Using Network Coding)	Using XOR NC on P2P Overlay intelligently which improves the throughput and decreases the point-to-point delay
**[47]**	Inter Street Interference Cancelation in Urban Vehicular Networks Using Network Coding 2014	Intervention omission through Multi Hop Beacon Forwarding and XOR Network Coding	The Increase in throughput and the reduction in point-to-point delay using XOR NC intelligently
**[22]**	Structured P2P Overlay of Mobile Brokers for Realizing Publish/Subscribe Communication in VANET 2015	Creation of a Structured P2P Overlay on VANET and prediction for the proper node selection through route table and hash table	Creating a P2P Overlay on VANET and performing XOR NC intelligently to increase the throughput and decrease the delay

## Preliminary

As the proposed method uses AHP and TOPSIS methods, this section defines them in details.

### AHP method

AHP is an analytic hierarchical process for multi-criteria decision-making which has been presented by Saaty [50]. AHP turns the issue of complex multi-criteria decision making into a hierarchy of decision elements, that is, the purpose, criteria, and decision options associated with that problem. AHP places criteria and options in the form of a hierarchical structure similar to a family tree. The first step is to draw the selection pattern.

**Fig 2:**
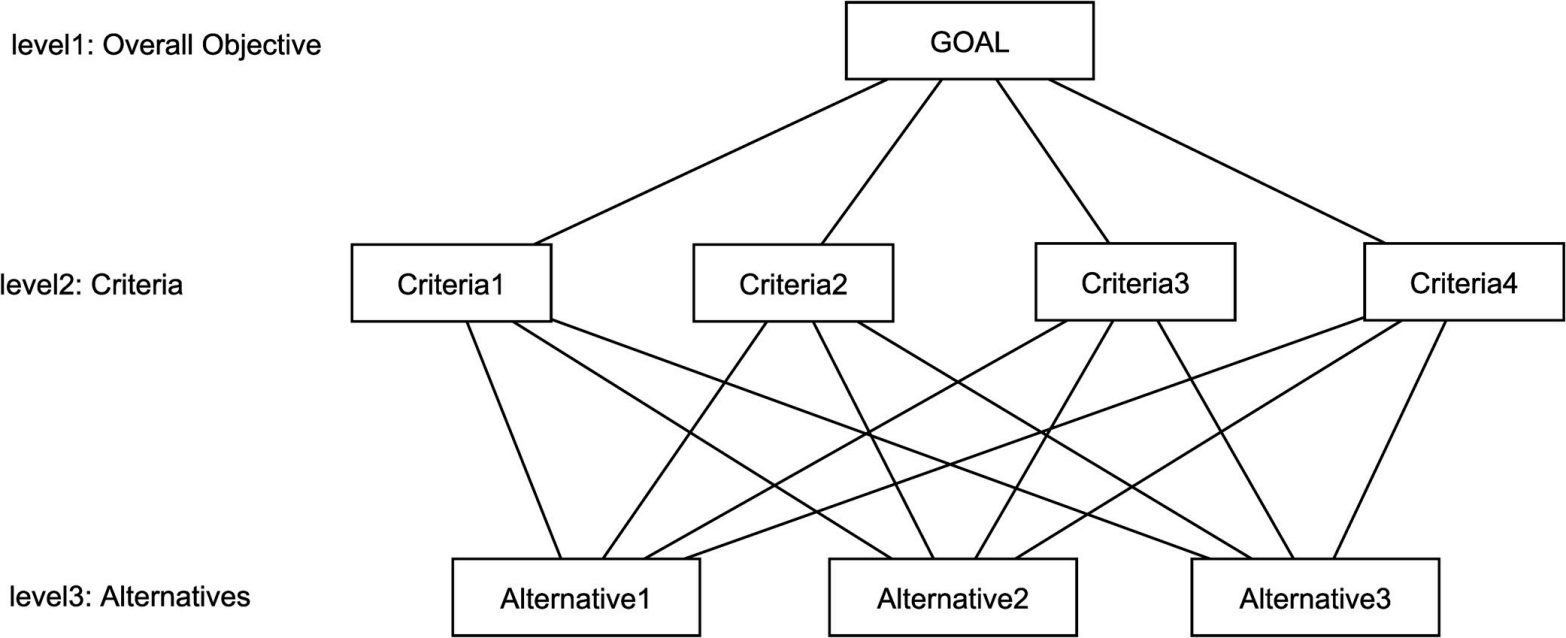
Hierarchical structure of AHP.

The hierarchy has at least three levels: the overall objective of the problem is placed in the first level, the multiple criteria that evaluate options are placed in the middle and decision options or alternatives are at the bottom. A sample representation of the decision-making hierarchy is depicted in Fig 2. The second step is to compare the options and criteria. When the problem is decomposed and its hierarchy is built, prioritization procedures begin to determine the relative importance of criteria in each level. Paired judgments begin in the second level (criteria) and end in the final level. In each level, the criteria are compared two by two based on their influence level and the specified criteria in the higher level.

Paired comparison should be made through asking questions from the decision maker. For example, we can ask, regarding the purpose of the decision, which scale 1 to 9, as shown in Table 2, should be allocated as the amount of the importance of the criteria? Also, the numbers 2,4,6,8 should be used to modify the comparison.

**Table 2:** The scale of paired comparisons in AHP method [51].

Numeral classification	Intuitive judgement
1	Same preference
3	Moderate preference
5	Strong preference
7	Very strong preference
9	Extreme preference
2،4،6،8	Moderate values

The table of the scale of paired comparisons in AHP is completed such as Table 3. In this table, Pc (ai,aj) represents the preference rate of ai criterion over aj. As shown, the preference in the same two criteria equals 1. This table contains a two-way feature, that is, aji = 1/aij. This table is used to compare the criteria and options [40].

**Table 3:** Paired comparisons [40].

C	a_1_	…	a_j_	…	a_n_
a_1_	1				
…		1			
a_i_			Pc(a_i_, a_j_)		
…				1	
a_n_					1

To achieve the weights, one should do the following [51]:

First, normalization is done through Eq 1:

$$a_{ij}^{*}=a_{ij}/\sum_{i=1}^{n}a_{ij}\;\forall j,j=1,2,\ldots ,n$$ (1)

In order to normalize, each element must be divided by the sum of all the elements of the same column from the paired comparison matrixes. Then, weights are calculated from Eq 2:

$$w_{i}=\sum_{i=1}^{n}a_{ij}^{*}/n\;\forall i,i=1,2,\ldots ,n$$ (2)

### TOPSIS method

TOPSIS was first introduced by Hwang. [52] The only mental data required by the TOPSIS method is the importance of the weights of the criteria, which has attracted the decision makers. In TOPSIS, the chosen option must have the least distance from the positive ideal solution and the most distance from the negative one [53]. Therefore, the TOPSIS concept requires the identification of an ideal point. Determining the ideal point is usually the first step in solving MCDM problems. In spite of having an ideal point, MCDM problems can be solved by options placement or decisions, depending on which one is closer to the ideal point.

Now the problem is how this distance is measured from the ideal point. TOPSIS uses the Euclidean method to calculate distances from positive and negative ideal points[54]. In the first step, a decision matrix is constructed for n options and m criteria. This matrix indicates the value of each option based on each criterion as x_ij_.

$$X_{n\times m}=\left[\begin{array}{ccc} x_{11} & \cdots & x_{1m}\\ \vdots & \ddots & \vdots \\ x_{n1} & \cdots & x_{nm} \end{array}\right]$$

In the next step, the importance of the weights is specified for each criterion, in a way that:

$$\sum_{j=1}^{m}w_{j}=1\;,\;j=1,2,\ldots ,m$$ (3)

Then, to descale the decision-making matrix, the division of each element of the decision matrix (xij) into the square of the total number of the squares of the values is used:

$$r_{ij}=\frac{X_{ij}}{\sqrt{\sum_{i=1}^{n}x_{ij}^{2}}}$$ (4)

The following matrix is the unscaled matrix of the problem:

$$R_{n\times m}=\left[\begin{array}{ccc} r_{11} & \cdots & r_{1m}\\ \vdots & \ddots & \vdots \\ r_{n1} & \cdots & r_{nm} \end{array}\right]$$

In the next step, the specified weights for each criterion are gained through multiplying by the unscaled matrix and unscaled weighted matrix:

$$V=W_{n\times n}.R_{n\times m}=\left[\begin{array}{ccc} V_{11} & \cdots & V_{1m}\\ \vdots & \ddots & \vdots \\ V_{n1} & \cdots & V_{nm} \end{array}\right]\;;\;W_{n\times n}=\{w_{1},w_{2},\ldots ,w_{n}\}$$ (5)

Now the positive ideal solution and the negative one can be calculated.

$$A^{+}=\left\{(i^{{max}_{v_{ij}}}|j\in B),(i^{{min}_{v_{ij}}}|j\in C)\right|i=1,2,\ldots ,n\}=\{v_{1}^{+},v_{2}^{+},\ldots ,v_{j}^{+},\ldots ,v_{n}^{+}\}$$ (6)

$$A^{-}=\left\{(i^{{min}_{v_{ij}}}|j\in B),(i^{{max}_{v_{ij}}}|j\in C)\right|i=1,2,\ldots ,n\}=\{v_{1}^{-},v_{2}^{-},\ldots ,v_{j}^{-},\ldots ,v_{n}^{-},\}$$ (7)

In the next step, Euclidean method is used to calculate the distance between the i-th solution and positive ideal solution (S_i_^+^) and negative one (S_i_^-^).

$$S_{i}^{+}=\sqrt{\left\{\sum_{j=1}^{n}{\left(v_{ij}-v_{j}^{+}\right)}^{2}\right\}},\;i=1,2,\ldots ,n$$ (8)

$$S_{i}^{-}=\sqrt{\left\{\sum_{j=1}^{n}{\left(v_{ij}-v_{j}^{-}\right)}^{2}\right\}},\;i=1,2,\ldots ,n$$ (9)

Finally, the relative closeness of i-th solution to the ideal solution is calculated through the division of the distance of that solution from the negative ideal one by the total distance of that solution from the negative and positive ideal solutions.

$$C_{i}^{*}=\frac{S_{i}^{-}}{\left(S_{i}^{-}+S_{i}^{+}\right)};\;0\leq T_{i}\leq 1;\;i=1,2,\ldots ,n$$ (10)

The best solution is the one with a higher index. Therefore, based on this index, the solution ranking is done [31].

### Hybrid AHP-TOPSIS approach

Different steps of AHP-TOPSIS is described below [41,52]:

1. To determine the appropriate criteria and to complete the matrix of the paired comparisons between them.
2. To draw a hierarchical structure for criteria and various options.
3. To get the weight of the criteria using AHP.
4. To rank and select the best combination using TOPSIS.

## Problem statement

Previous researches show that XNC is an efficient and practical method for video broadcasting in vehicular networks. According to these studies, although XNC increases the throughput considerably [33,38,39,45,55,56], it suffers from the following problems:

1. Due to the existence of a communication structure in a vehicular network, XNC is not efficient with regard to the broadband consumption rate. The key idea to improve this method discusses the reduction of unnecessary codes dissemination while a code-receiver node should be able to decode and extract a large number of required frames.
2. The increase in the point-to-point delay is caused due to the lack of attention to the neighbor’s buffer status and sending several coded packets to empower them to decode the required frames. In other words, the traffic communication is increased and consequently, point-to-point delay is increased, also.

Therefore, is there any efficient solution to decrease the effects of the above-mentioned problems with regard to the neighbour’s buffer status and as a result, the reduction in the number of exchanged messages, while protecting the efficiency of XNC in live video broadcasting system in vehicular networks?

The next section will propose a solution to answer this question and solve the mentioned challenges for providing high video quality on vehicular.

## The proposed method

It is thought that broadcasting carries out a bitwise XOR operation on the packets which are received before relaying them and is capable of forwarding the packets containing the information of average data rates of sources. Broadcasting makes a queuing buffer continue. The BSCS (Buffer Size Control Scheme) intends to check the imposed delay by checking the buffer size. The TCS (Time Control Scheme) determines a delay limit for each packet, and this leads to the fact that the packet cannot be buffered more than a specific Tmax time.

In transferring the live video packets in vehicular networks, to improve the congestion control, increase the throughput amount, reduce the point-to-point delay, and receive the video with a high quality, XNC is intelligently used by this research. Therefore, the nodes should be aware of their neighbours buffering map and combine packets in a way that other network vehicles can easily obtain their desired data. In this proposed method, an unstructured P2P Overlay is created on VANET network. P2P Overlay networks make each network more flexible, widespread and expandable. In the unstructured model, there is no stable topology and peers’ connections are created arbitrarily which in turn, is better for video broadcasting.

Then, each RSU, using the buffer map of both RCUs which are connected to it and vehicles which are under its coverage, will learn about both available and required packets in them. Then, the best packet combination is selected through AHP or AHP-TOPSIS, XNC is performed and broadcasted. Therefore, network vehicles can easily achieve their desired packets with the help of a higher chance. Because of that, a lower number of packets are transferred through the network. Also, the throughput is increased and the delay is decreased.

Consequently, the quality of the received live video is improved in vehicles. This is a novel combination of VANET, P2P Overlay, and XNC network. When coding combinations are created, they are rated and prioritized (intelligently). While rating them:

1. First, an important frame such as I frame, whose broadcasting time is closer, should be considered.
2. Second, important frames such as P whose broadcasting time is closer, should be considered.
3. Third, unimportant frames such as B whose broadcasting time is close, should be considered.
4. Fourth, important frames such as I and P whose broadcasting times are not close, should be considered.
5. Fifth, unimportant frames such as B whose broadcasting time is not close, should be considered.
6. Sixth, frames which are possibly going to be decoded and will be used in the far future should be considered.

Some expressions are explained in Table 4.

**Table 4:** Defining some of the expressions which have been used.

Expression	Definition
RSU	Stable roadside equipment through which the vehicle is connected to the communicational platform. Usually RSU is the host of an application that provides services
OBU	OBU is a peer-to-peer device that uses the services provided. Each vehicle has an OBU and a set of sensors which collect and process the information and send it as a message to other vehicles or RSUs via wireless media.
xij	The size of the value of i based on j index
rij	The unscaled value in the decision making matrix
vij	The unscaled weighted value for i in j index
A^+^	The positive ideal solution is the highest amount of a solution in positive criteria (benefit-based criteria) or the least amount of that solution in negative criteria (cost-based criteria) in a weighted scalar matrix
A^-^	Negative ideal solution is the least amount of solution in positive criteria or the highest amount of solution in negative criteria in weighted scalar matrix.
B	B is a set of criteria with positive semantic load (such as benefit)
C	C is a set of criteria with negative semantic load (such as cost).
Buffer map	a message that contains the offset (number of the first piece), the buffer map length, and a string of zeros and ones that indicates which piece is available
Pause time	Each node stops for a predetermined time, the pause time (PT), and after that time, moves to the randomly selected point on a straight line. After reaching the destination, it again pauses for a specific PT and the same thing is repeated again to move to another point.

### Proposed method details

**Fig 3:**
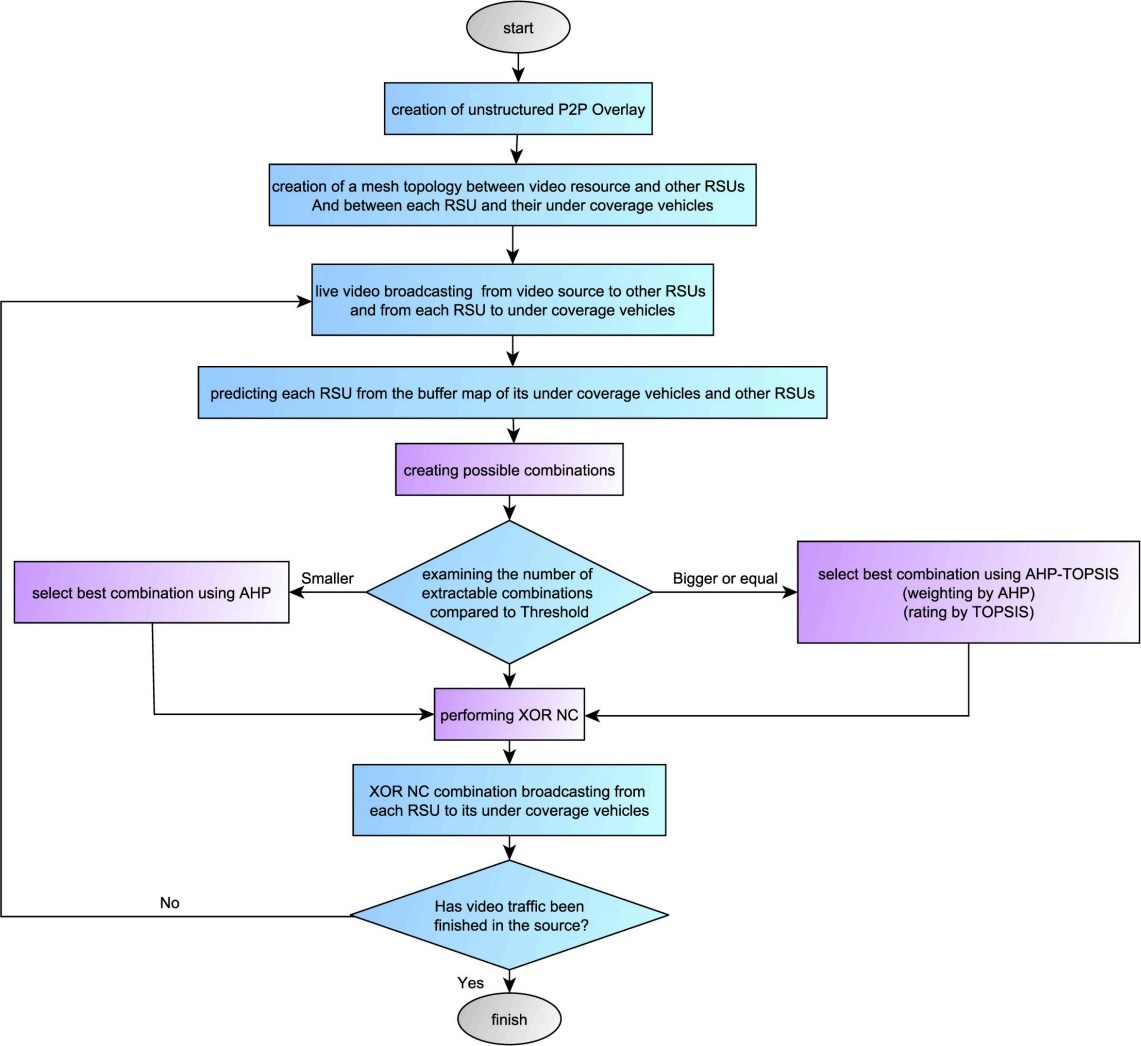
The proposed method on RSU side.

**Fig 4:**
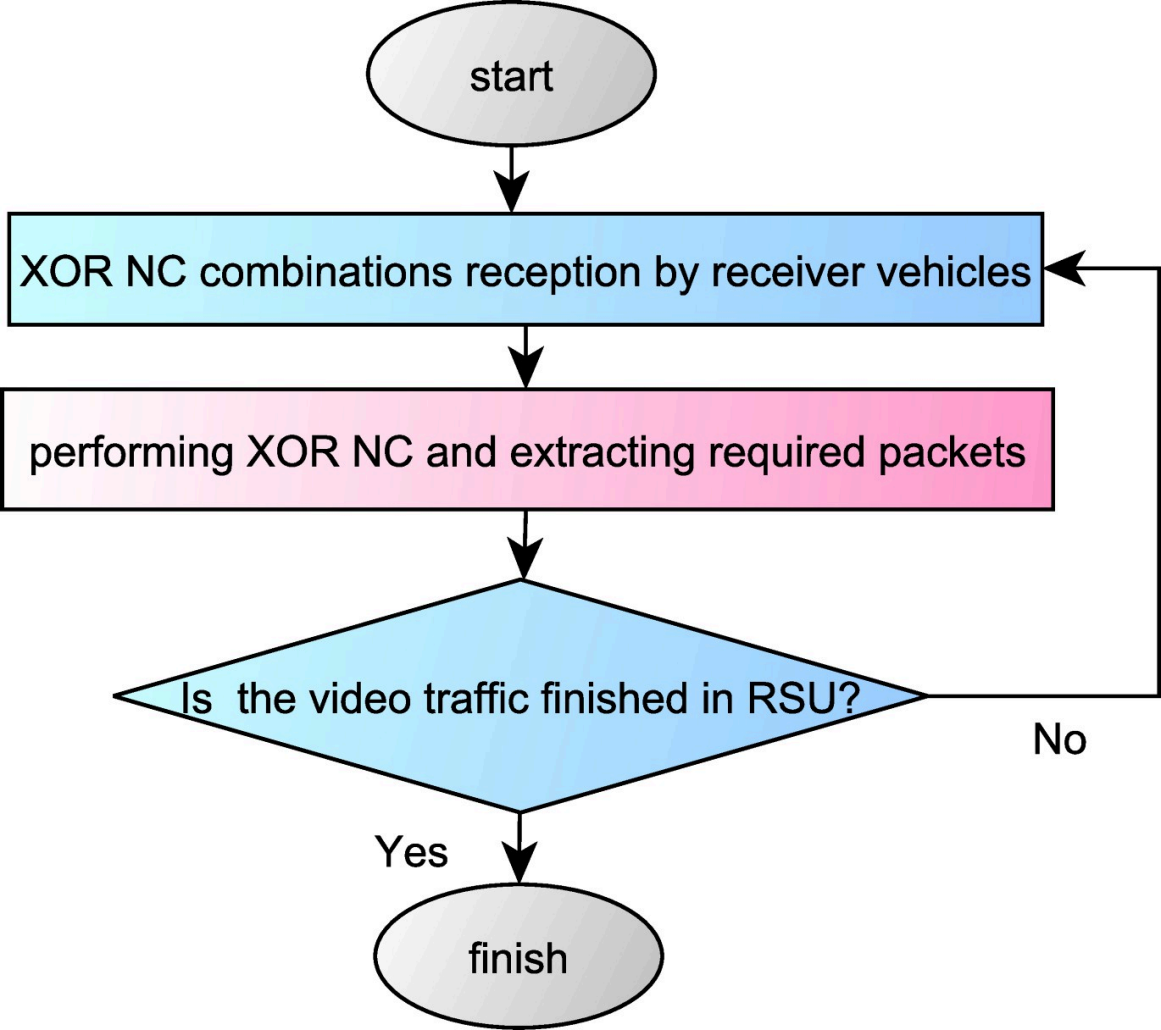
The proposed method in the receiver vehicles.

In Fig 3, a view of the suggested method is shown on the RSU side. Also, in Fig 4, this view is depicted from the side of receiver vehicles.

Because the video source broadcasts the video for all RSUs, a mesh topology is created between the video source and each RSU (Due to the high mobility of vehicles, the selection of tree topology leads to different divisions of the tree into smaller parts and this, in turn, increases costs). That is why a mesh topology is created between each RSU and its under coverage vehicles. The live video is broadcasted from the video source to each RSU and from each RSU to its under coverage vehicles according to GoP16 structure. The vehicles will buffer the video for the first 10 seconds and then start broadcasting it.

Under coverage vehicles of each RSU and RSUs connected to that send their buffer map to the RSU alternately (for the number of simulation runs). As a result, RSU is able to learn about the buffer map of its under coverage vehicles and to examine what packets they have and what packets they need. It can also learn about the buffer map of RSUs which are connected to it. In addition, it can learn about the number of its required frames which other RSUs possess, to be able to have the best combination for packet XORing.

**Fig 5:**
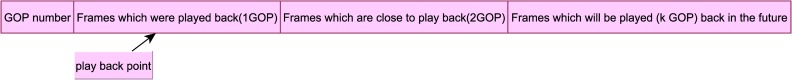
Vehicles buffer map.

In Fig 5, the buffer map of each vehicle is depicted. The first section contains the number of the last played back GoP. The second part includes the status of the current playing back GoP in a sequence of ones and zeros. The next two GoPs that are close to playback are shown as one and zero bits in the third section. The number “one” represents the existence of frames and “zero” represents lack of frames. As a result, it can be learned that what frames the vehicles have or need to play the video. The last section contains the frames which will be played back in the future including K GoPs. The number of these frames is defined dynamically and is changed according to the network traffic. With regard to available fames as well as required ones in each RSU and their under-coverage vehicles, a series of XOR combinations could be made of the required frames using permutation formula (n!). The number of extractable combinations is surveyed by comparison with the Threshold (the number that is selected by the simulator dynamically and is changed along with the momentary changes of the network).

The proposed method for selecting the best packets combination is AHP. Due to AHP’s limitation in the number of combinations, the increase in the number of combinations leads to the increase in AHP’s complications. When the number of combinations exceeds the Threshold, AHP-TOPSIS method is used for choosing the best combination. The performance of AHP and AHP-TOPSIS is explained in Example 1.

In this article, XNC is performed intelligently. That is, in each transmission the best combination is selected (frame selection is done by AHP and AHP-TOPSIS based on their weight and priority). Therefore, to combine the XOR NC packets (with regard to the available information in node buffer and receivers buffer status), the proposed method should act in a way:

1. To have the highest possibility of decoding among the neighbors (in each node).
2. To deliver the important frames (firstly I and secondly P) to the receivers quickly.
3. To reduce the lost (the times used to broadcast a video should not be wasted).

Also, to have a video received with a higher quality, the following order is used for criteria priority:

1. Close to play back,
2. Frames priority,
3. Highest possibility of being decoded.

When the best frame combination is chosen, the available frames in that combination are XoRed and transmitted to the vehicles under coverage of each RSU in the form of one packet. Under coverage vehicles of each RSU receive XOR NC combinations. To broadcast the video with a higher quality, vehicles can achieve their desired frames through using XOR NC for the second time.

#### Example 1

**Fig 6:**
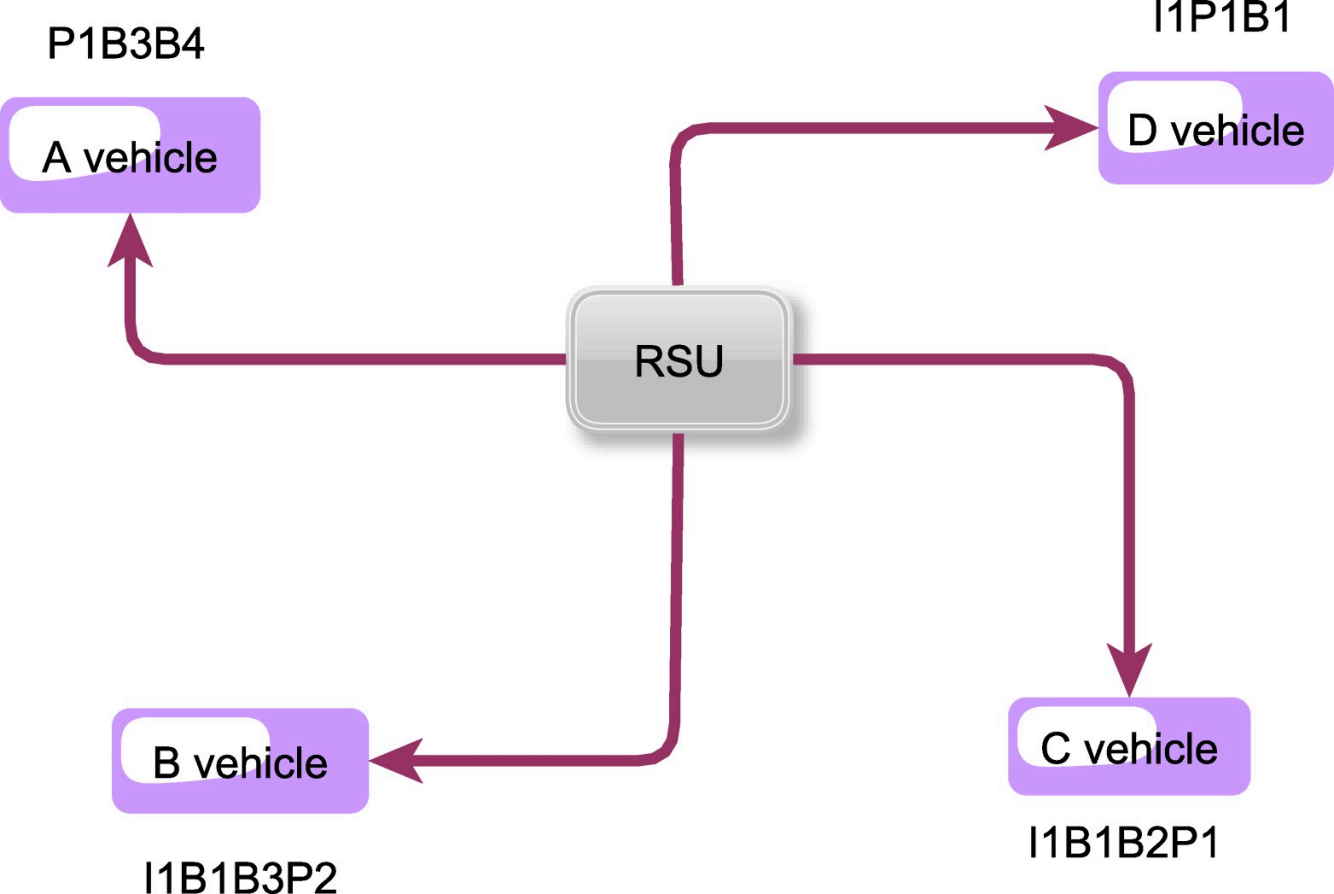
The structure of Example 1.

We consider a RSU which wants to send the information for 4 other vehicles. It should transmit the best combination for its neighbors. We assume that all frames are I1B1B2P1B3B4P2 and RSU contains I1P1B3B4P2 frames. In their own buffer maps, each vehicle has the frames which are shown in the following figure (Fig 6).

Through the buffer map of each vehicle, one can learn what frames each vehicle needs. Then, using AHP or AHP-TOPSIS, the best combination can be chosen. Later, network coding is done and sent to each vehicle. As it is shown in the figure, vehicle A needs I_1_B_1_B_2_P_2_ frames, vehicle B needs B_2_P_1_B_4_, vehicle C requires B_3_B_4_P_2, and vehicle D needs_ B_2_B_3_B_4_P_2_ frames.

### Solving an example through using AHP

**Fig 7:**
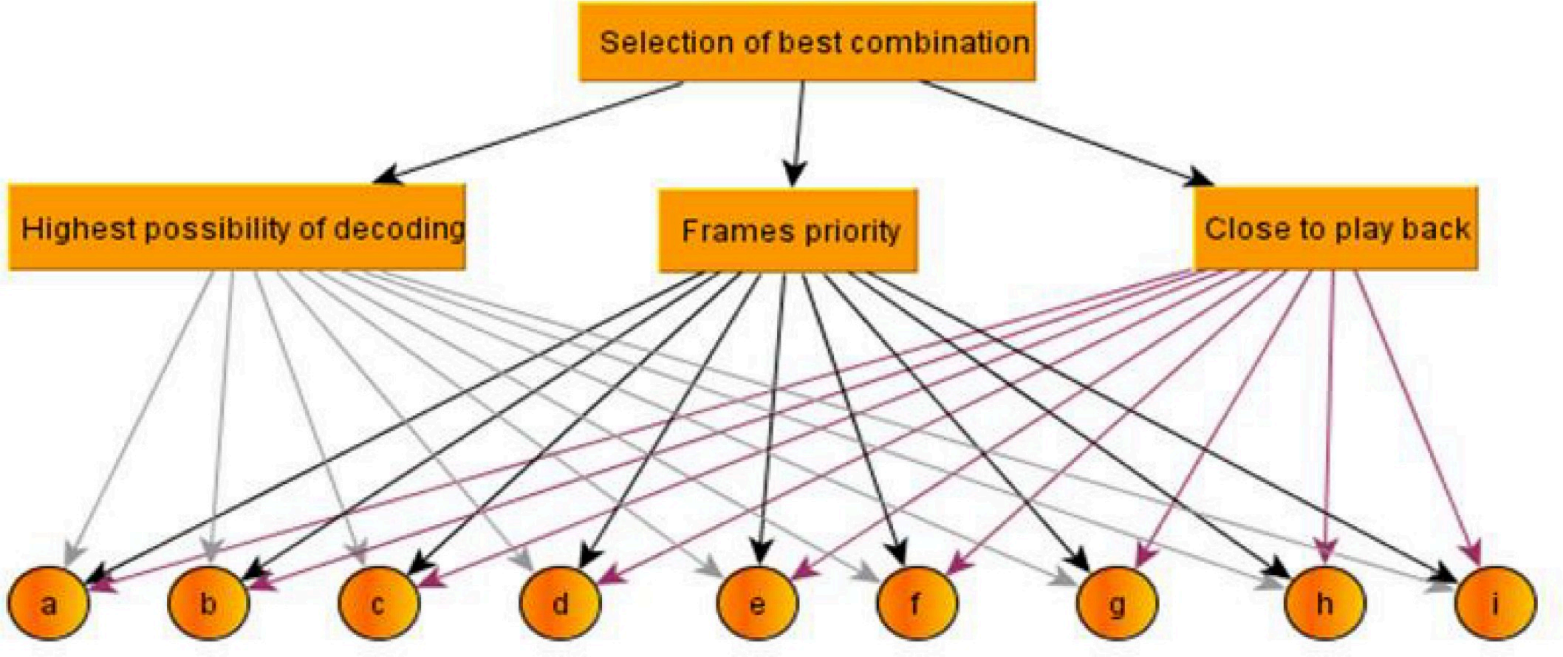
Selection pattern drawing.

In the beginning, the goal is to choose the best combination. Then, criteria and candidates are chosen. Candidates are defined as the number of possible combinations made of required frames of vehicles, which is achieved through permutation formula. Then, such as Fig 7, the selection pattern is drawn.

Among the possible combinations, four of them are chosen as samples to be tested through AHP and AHP-TOPSIS in order to reach the best combination. In the next step, solutions are compared two by two based on the criteria and are given priority over each other.

We should get the geometric mean of each row. The geometric mean is obtained from nth root of a product of n numbers. Then the total geometric mean of all rows is calculated. Normalization is done by dividing the geometric mean of each row to the total geometric mean of all rows and the numbers are normalized. For each table, comparison, geometric mean and normalization are performed. In Tables 5–7, the combinations are chronologically compared two by two based on the following criteria: being close to be played back, frames priority and the highest possibility of decoding.

**Table 5:** Paired comparisons of combinations based on being close to play back-criterion.

Normalized	Geometric mean	i	h	c	A	Close to play back
0.044	0.253	1/7	1/7	1/7	1	a
0.42	2.432	5	1	1	7	c
0.42	2.432	5	1	1	7	h
0.116	0.669	1	1/5	1/5	5	i
1	5.786					

**Table 6:** Paired comparison of combinations based on frames priority-criterion.

Normalized	Geometric mean	i	h	c	a	Frames Priority
0.045	0.293	1/3	1/9	1/5	1	a
0.204	1.316	3	1/5	1	5	c
0.654	4.213	7	1	5	9	h
0.095	0.615	1	1/7	1/3	3	i
1	6.437					

**Table 7:** Paired comparison of combinations based on decodable-criterion.

Normalized	Geometric mean	i	h	c	a	Decodable
0.545	2.817	3	7	3	1	a
0.193	1	1	3	1	1/3	c
0.069	0.355	1/3	1	1/3	1/7	h
0.193	1	1	3	1	1/3	i
1	5.172					

As before, in Table 8, the criteria are compared and ranked two by two according to the goal.

**Table 8:** Paired comparison of criteria in this order (1. Close to be played back 2. Frames priority 3. Being decodable).

Normalized	Geometric mean	Decodable	Frames priority	Close to play back	Status
0.715	3.271	7	5	1	Close to play back
0.218	1	5	1	1/5	Frames priority
0.067	0.306	1	1/5	1/7	Decodable
1	4.577				

Table 9 contains the normalized geometric mean of each combination based on each criterion.

**Table 9:** The normalized geometric mean of combinations based on the criteria.

Decodable	Frames priority	Close to play back	Decodable
0.545	0.045	0.044	a
0.193	0.204	0.42	c
0.069	0.654	0.42	h
0.193	0.095	0.116	i

To calculate the priorities, we can get the weight of each option. To achieve that, the sum of the product of the multiplication of the priority of that option on the basis of the criterion is multiplied by its priority; that is, for each combination in Table 8, we multiply the columns which are related to a combination on the basis of criteria by the priority of that criterion in Table 7. The option which gets the highest points will be chosen as the best combination. Here, h is the best one.

a = 0.044 *0.715 + 0.045 * 0.218 + 0.545 * 0.067 = 0.076

c = 0.42 * 0.715 + 0.204 * 0.218 + 0.193 * 0.067 = 0.353

h = 0.42 * 0.715 + 0.654 * 0.218 + 0.069 * 0.067 = 0.448

i = 0.116 * 0.715 + 0.095 * 0.218 + 0.193 * 0.067 = 0.11

### Solving the example using AHP-TOPSIS method

To solve the example using AHP-TOPSIS, as with the AHP method, first we set the tables of paired comparisons of combinations based on the criteria and the paired comparison table of criteria. Then we multiply the criteria priority which was calculated in Table 7, by the drawn matrix in Table 9 to reach Table 10.

**Table 10:** Normalized geometric mean of combinations based on the criteria.

Decodable	Frames priority	Close to play back	Decodable
0.036	0.009	0.031	a
0.013	0.04	0.3	c
0.005	0.143	0.3	h
0.01	0.02	0.08	i

In this table, we calculate the positive and negative ideal solutions. The maximum and minimum amounts are calculated in each column. Then all maximums and minimums are put together in one row.

- Min = Worst Alternative = (0.031, 0.009, 0.005)
- Max = Best Alternative = (0.3, 0.143, 0.036)

For each combination, the distance between minimums and each single row and also, maximums and each single row is calculated. Here, for a, c, h, and i combinations, the best and worst solutions are specified.

$$\begin{array}{l} \mathrm{Best}=\sqrt{{\left(0.3-0.031\right)}^{2}+{\left(0.143-0.009\right)}^{2}+{\left(0.036-0.036\right)}^{2}}=0.3\\ \mathrm{Worst}=\sqrt{{\left(0.31-0.31\right)}^{2}+{\left(0.009-0.009\right)}^{2}+{\left(0.036-0.005\right)}^{2}}=0.031 \end{array}$$ a

$$\begin{array}{l} \mathrm{Best}=\sqrt{{\left(0.3-0.3\right)}^{2}+{\left(0.143-0.04\right)}^{2}+{\left(0.036-0.13\right)}^{2}}=0.107\\ \mathrm{Worst}=\sqrt{{\left(0.3-0.031\right)}^{2}+{\left(0.04-0.009\right)}^{2}+{\left(0.013-0.005\right)}^{2}}=0.073 \end{array}$$ c

$$\begin{array}{l} \mathrm{Best}=\sqrt{{\left(0.3-0.3\right)}^{2}+{\left(0.143-0.143\right)}^{2}+{\left(0.036-0.005\right)}^{2}}=0.031\\ \mathrm{Worst}=\sqrt{{\left(0.3-0.031\right)}^{2}+{\left(0.143-0.009\right)}^{2}+{\left(0.005-0.005\right)}^{2}}=0.3 \end{array}$$ h

$$\begin{array}{l} \mathrm{Best}=\sqrt{{\left(0.3-0.08\right)}^{2}+{\left(0.143-0.02\right)}^{2}+{\left(0.036-0.01\right)}^{2}}=0.066\\ \mathrm{Worst}=\sqrt{{\left(0.08-0.031\right)}^{2}+{\left(0.02-0.009\right)}^{2}+{\left(0.01-0.005\right)}^{2}}=0.056 \end{array}$$ i

Now, we select the solution which has the longest distance from the worst to be the best solution. In this method, h has the longest distance and is the best combination.

$$a=\frac{0.031}{0.031+0.3}=0.094$$

$$c=\frac{0.073}{0.073+0.107}=0.405$$

$$h=\frac{0.3}{0.3+0.031}=0.906$$

$$i=\frac{0.056}{0.056+0.066}=0.459$$

The proposed method in this article is based on the reference number [26]. The considered variables include the number of vehicles and the mobility of vehicles, whose effect on the amount of the throughput and point-to-point delay is examined.

## Evaluating the results

### Means and parameters of simulation

To do the simulation tests, the combination of NS2 and MobiSim (vehicular simulator), which are related to vehicular networks, is used.

- The NS2 software is simply a discrete-event simulation tool developed in 1989 by the University of California and the Cornell University that created the real-network simulator and is used to study the dynamic nature of communication networks. The simulator NS2 has the following characteristics:
- Scenario Production: This feature enables the user to evaluate their protocol in various conditions. The scenario production involves automated network connectivity, traffic patterns, and dynamic events. The automatic scenario production plays an important role in the systematic testing of the protocol.
- Development capability: Adding new library functions and developing available protocols in the NS-2 can easily be done without changing other protocols. Designing new protocols with a C scripting language is done in an object-oriented way.

This simulator has been popularized and permanently used in research communities because of its flexibility and modular nature [57]. MobiSim software is also used to simulate roads and geo-routing of transport in urban space scenarios in Ad-hoc networks. This software is used in Linux operating system under Java open source [57]. In Table 11, simulation parameters are stated.

**Table 11:** Simulation parameters.

Parameter	Quantity	Description
Radio range of the node	250 meters	In all simulations, 250-meter radio range nodes are used
Network dimensions	1000*1000	Nodes are placed in a 1000 by 1000- meter space randomly
Node movement model	random waypoint	In all performances, random waypoint model is used for node mobility in the network. In this model, each node selects a point randomly as a destination, then with a moderate speed which is between max and min speed moves toward the destination. When the node reaches the destination, it stays there for some time which is called the pause time and then, it repeats this action again.
Noise model	Gaussian random model	In all performances, Gaussian random model is considered.
Simulation time	3000 seconds	The needed time for each simulation is 3000 seconds. Each of the recorded results is an average of 100 times performing the scenario
Min speed	5 meters per second	The speed of vehicle
Max speed	60 meters per second	The speed of vehicle
Node number	200	available vehicles
Physical layer	MAC	802.11 media access protocol
Traffic type	VBR	in all scenarios, VBR traffics are randomly imposed
Packet size	512 bytes	The size of each packet is considered as 512 bytes. Each node decides to transmit the information and transmits 25 packets per second for each destination.
Primary power of each node	2 Joules	In all simulations, we use the nodes with 2 joules primary power
size of GoPs	G16B1	In the simulation, 16 framed GoP have been used and between P and I frame, there is only one B frame, which is G16B1.
movie name	Star War IV (G16B1, Single Layer)	The movie used in this simulation was Star War IV.
QP (Quantization Parameter)	8	The amount of image compression

### Evaluation criteria

- **The throughput**

Throughput is the packets which are transmitted safely in a rate of time and is got through the following relation:

$$\mathrm{Throughput}=\frac{\mu }{t}$$ (11)

In this relation, μ is the number of received bits in a time unit (t). (For the whole network, it equals the average of throughput divided by total number of nodes)

$$PDR=\frac{Receive\_Pckt}{Sent\_pckt}$$ (12)

Packet delivery rate (PDR) is the packets which are delivered safely, while throughput is the delivered safe packets with the rate of speed in a specific time.

- **End-to-End delay (EED)**

The amount of time that each packet averagely needs to go from the origin to the destination is called end-to-end delay, and is calculated through the following relation:

$$D=T_{d}-T_{S}$$ (13)

In this relation, D is the delay time of a packet, T_d_ is the reception time of a packet in destination and T_s_ is the transmission time of a packet in origin.

In this section, the proposed algorithm is compared to BSCS and TCS algorithms with regard to the average end-to-end delay versus the maximum speed of nodes, pause time of nodes, and the number of nodes. Also, the throughput amount is examined versus the maximum speed of nodes and the number of nodes.

### Simulation results

In this section, we examine our method and compare it with others.

#### Point-to-point delay

**Fig 8:**
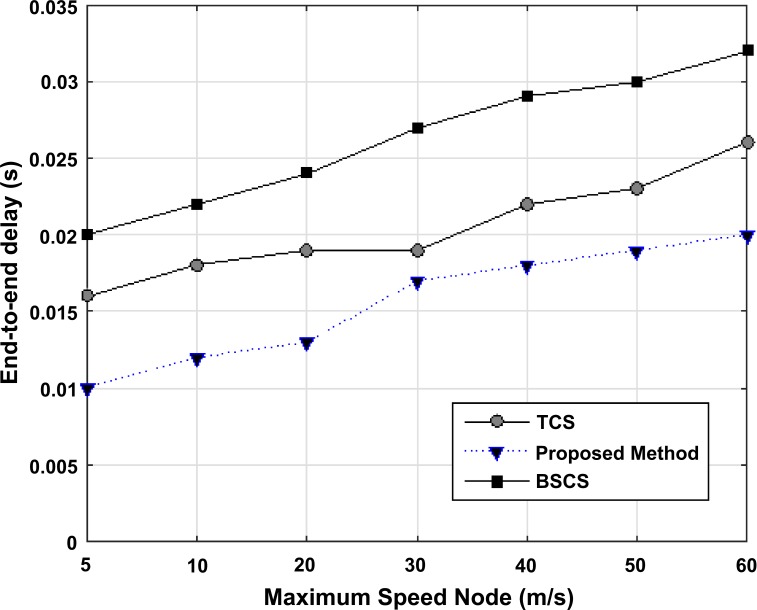
Point-to-point average versus the maximum speed of nodes in BSCS, TCS, and the proposed method.

The point-to-point delay of a packet is determined through the following parameters: leading conductivity delay, queuing delay, transmission delay, and packaging delay. As it is shown in Fig 8, the average of point-to- point delay versus the maximum speed of nodes is shown in three algorithms: BSCS, TCS and the proposed algorithm. In this simulation, the pause time is one second. By increasing the maximum speed of nodes in all three algorithms, the average point-to-point delay increases, due to the network topology dynamicity and the increase in the rate of routes breaking. The fact that the point-to-point average is lower in the proposed method compared to other algorithms is because of the efficient combination using AHP or AHP-TOPSIS methods. Therefore, fewer packets are transmitted in the network and nodes are easily able to reach their desired packets. Due to the use of random data and the number of simulation performances, various results are achieved whose average is shown on the graph.

**Fig 9:**
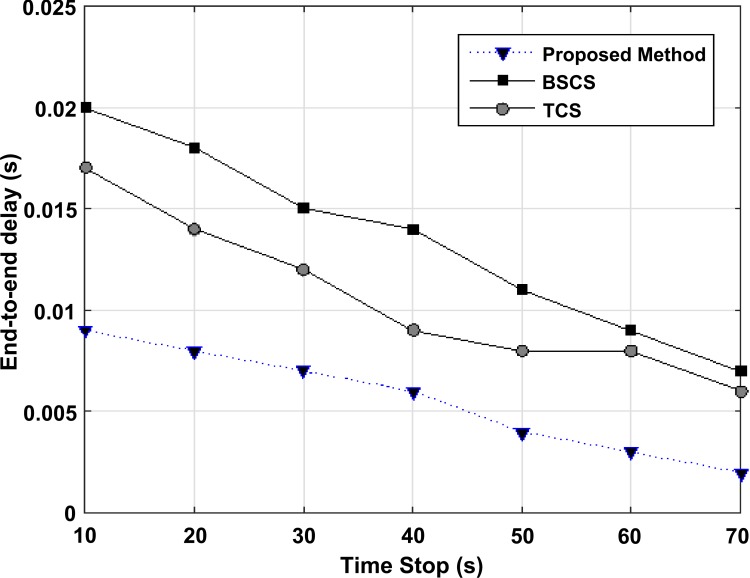
The average of point-to-point delay versus pause time of nodes in BSCS, TCS and the proposed method.

In Fig 9, point-to-point average versus pause time of nodes is compared in BSCS, TCS and the proposed method. In this simulation, the maximum speed of nodes is 25 meters per second and the number of nodes is 100. The data sample which is used in simulation is related to the H.264/AVC standard video coding. In this case, the pause time of nodes is changed. As it is shown, by increasing the pause time of nodes in simulation, the network dynamicity is decreased and the network turns into a more stable one and it causes rerouting to be stopped. In this case, the increase in the pause time of nodes decreases the point-to-point delay and it has higher slope in the proposed method. Because of having the best combination, XORing and transmission to the nodes through the packet is increased, and when nodes go towards stability, rerouting decreases and the links loss rate will be reduced. As a result, the point-to-point delay is decreased.

The simulation is performed to measure the vehicle's pause time until the vehicle reaches a static state. For example, the simulation is performed and the vehicles are stopped 20 seconds later; the results are reviewed and their average is calculated. As you can see in Figures, the chart shows that the vehicle has had more transaction with its own RSU. For example, at the pause time of 60 seconds, when the RSU and vehicles are much more connected, due to the location of the vehicles in the network and the transmission of RSUs, and the vehicles being set in the radio range with RSUs, we can have a lower end-to-end delay.

**Fig 10:**
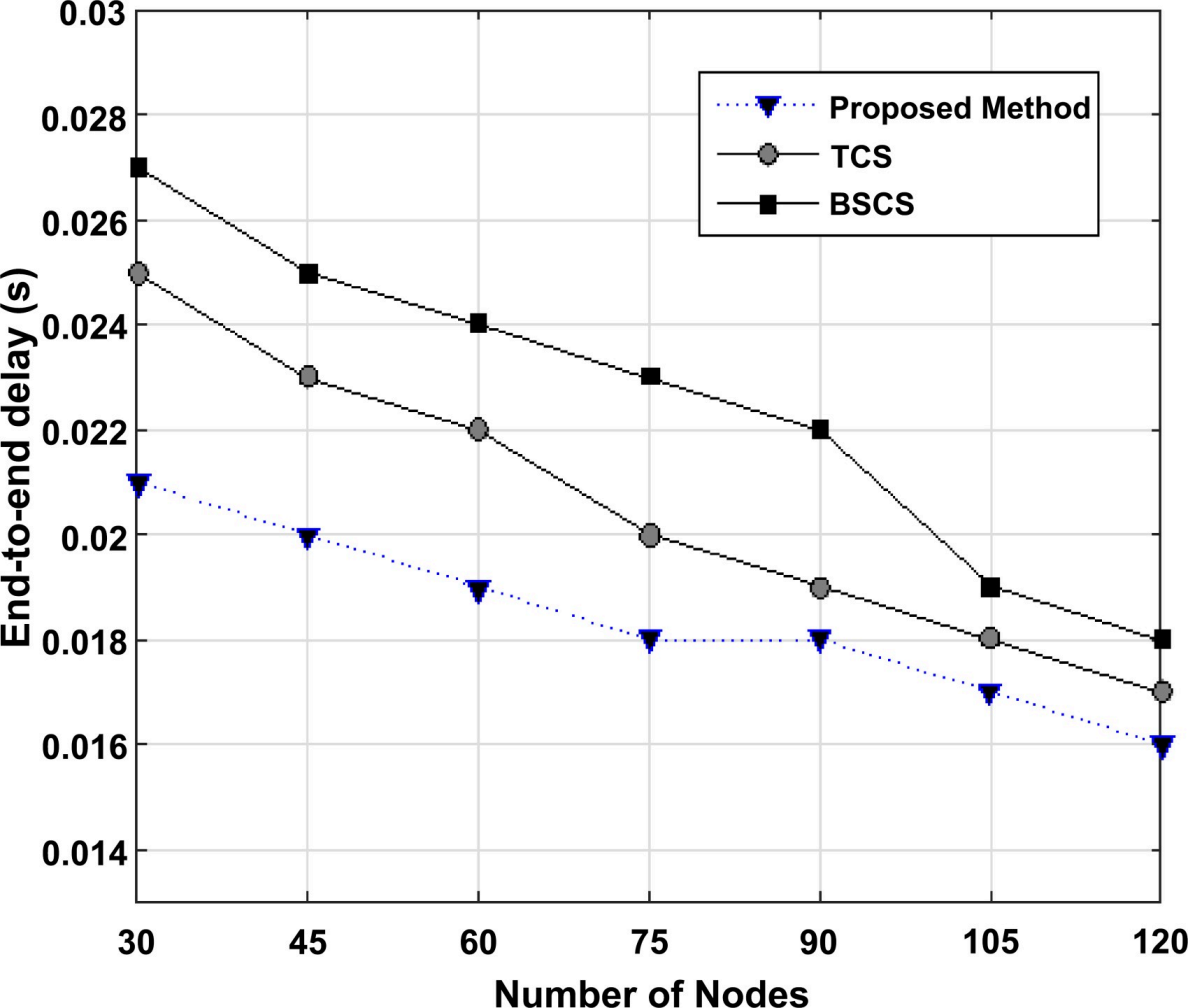
The average of point-to-point delay versus the number of nodes in BSCS, TCS, and the proposed method.

In Fig 10, three algorithms are examined with regard to the average of point-to-point delay versus the number of nodes, with a small difference; in this simulation, the maximum speed of nodes is 25 meters per second and the power of each node is 2 joules and the pause time is 1 second. In this case, the number of nodes changes.

As it is shown, the increase in the number of nodes does not have an intense influence on point-to-point delay. However, the increase in the number of nodes and in turn, the increase in the network density lead to the reduction in the average of point-to-point delay in all three algorithms, and actually this is because of the increase in the number of routes between the origin and destination.

#### The evaluation of throughput power

To calculate the throughput of the proposed method, in the input section of the node NS2 parameter, a counter is set, which sends the packets with the specified length in bytes for3000 times, at a different data rate which is byte per second. This information is moving between the specified nodes in the origin and destination. Each time the proposed method is performed in this case, the parts related to CRC (Cyclic Redundancy Check) and the transmitted frame ACK are inactive. As the results show, the change in data speed rate adjustment, can lead to a different throughput power in implementation system.

**Fig 11:**
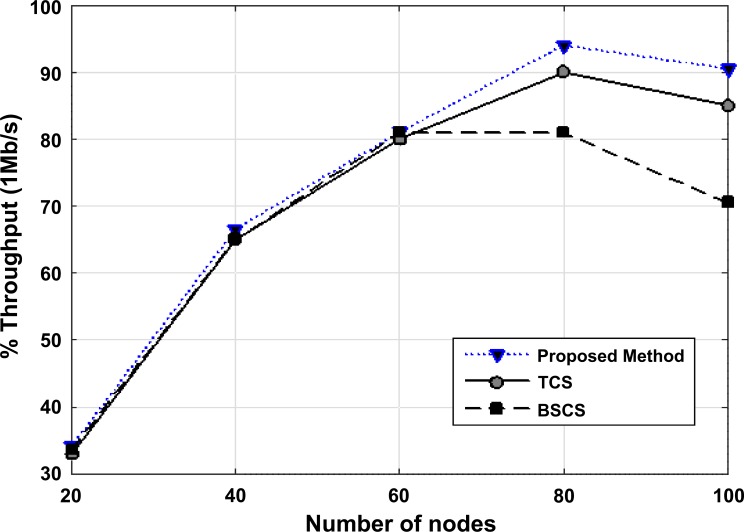
The throughput versus the number of nodes in BSCS, TCS, and the proposed method.

Fig 11 shows the throughput versus the number of nodes in BSCS, TCS and the proposed method. The change in the data rate of transmitter node results in a different throughput amount. The experiments have been done based on the number of repetitions (the number of repeated times for sending x byte) between the sender and receiver. By increasing the number of vehicles in the network, if the packet reaches the destination, the proposed method proves to be efficient. At the beginning of the simulation, when there is a smaller number of vehicles (up to 60 nodes), all three protocols act the same; but when the number of vehicles exceeds 60, the proposed method acts more efficiently due to utilizing the combined AHP-TOPSIS method.

According to the figure, due to network variations and the randomness of the nodes' environment in different conditions, for different speeds of the vehicle, various throughputs are observed. For example, at the speed of 20 m/s, all three algorithms have approximately the same throughput. When the speed changes (for example, at 30 m/s), network dynamics varies and also, the topology changes. Now we should observe if there is a radio range between the vehicle and the RSU in this situation at different speeds. At speeds of 50 and 60, due to the changes in the network dynamics and network topology, for broadcasting the vehicle randomly in the network, the throughput has changed. Throughput in the proposed method has improved and is better than the other two protocols.

**Fig 12:**
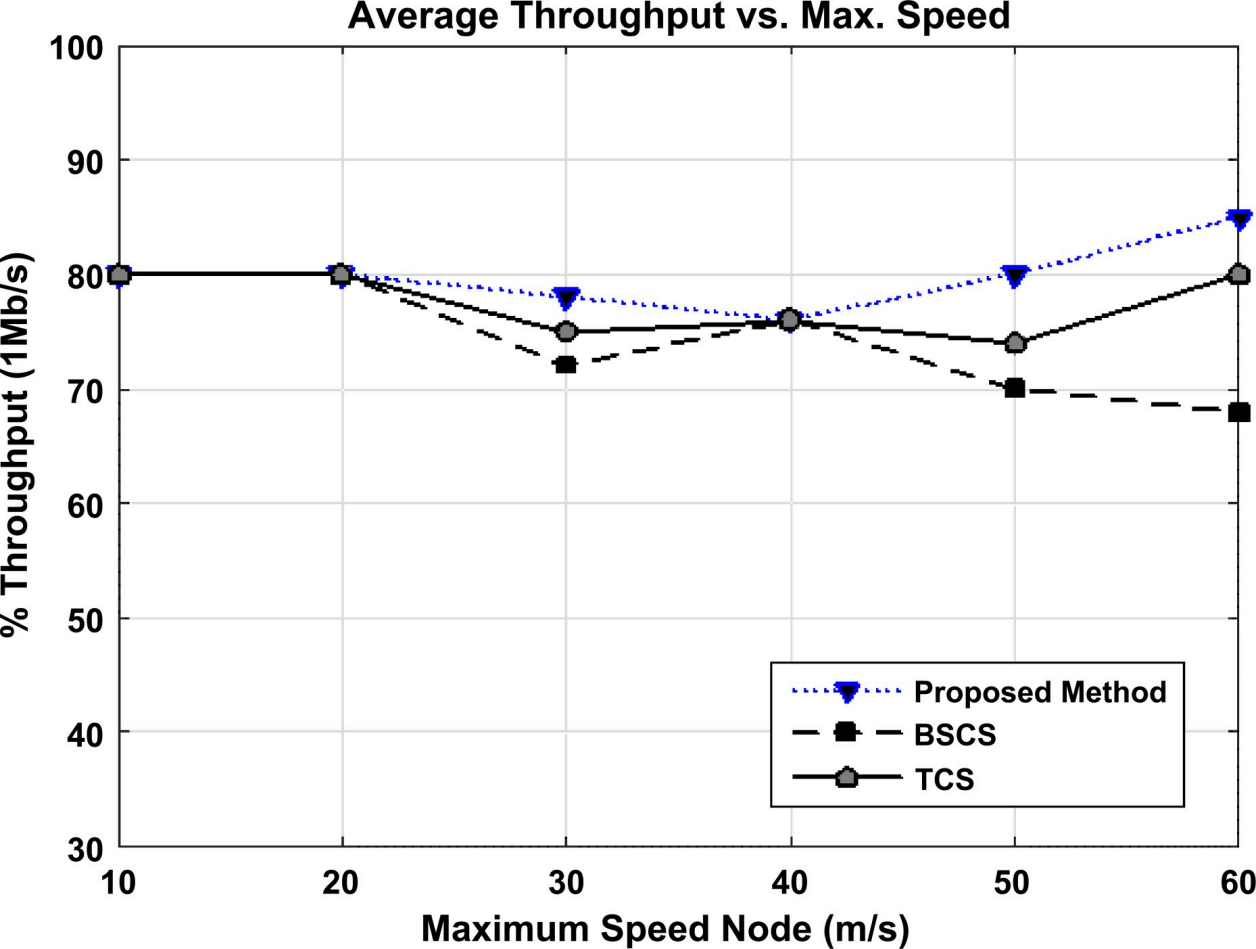
The throughput versus the maximum speed of nodes in BSCS, TCS, and the proposed method. In Table 12, the improvement percentage of the proposed method compared to BSCS and TCS is stated.

Fig 12 depicts the number of transmitted bits by the node and the number of received bits in the network at 1 megabit per second, regardless of the origin and confirmation packet. The increase in the maximum speed of nodes and information transfer speed has led to a considerable increase of throughput in the proposed method, compared to other methods.

**Table 12:** The improvement percentage of the proposed method compared to BSCS and TCS.

Parameter	Improvement percentage compared to BSCS	Improvement percentage compared to TCS
End-to-end delay average versus the maximum speed of nodes in all three algorithms	43 percent	17 percent
End-to-end delay average versus the pause time of nodes in all three algorithms	46 percent	23 percent
End-to-end delay average versus the number of nodes in all three algorithms	21 percent	12 percent
The throughput versus the number of nodes in all three algorithms	17 percent	8 percent
The throughput versus the number of nodes in all three algorithms	11 percent	5 percent

## Conclusion

There are some problems in transmitting the live video packets in vehicular networks such as the reduction in network congestion, efficient consumption of broadband, and the reduction in point-to-point delay. To remove these problems, network coding is used. The problem with XNC is that, when a node sends the packets for two other nodes, what the best combination of packets could be to enable other nodes of the network to use the broadcasted packet in the best way and to extract the highest amount of data. To do that, XNC is used intelligently, and using AHP and AHP-TOPSIS, the best combination of packets is selected and broadcasted in order to enable other vehicles of the network to reach their desired data. Therefore, fewer packets are transmitted in the network, the network congestion is decreased, the broadband is increased and point-to-point delay in mobile vehicular networks is reduced, and finally, the quality of received live video in vehicles is increased. More researches can be done in the future. For instance, this method can be done for VoD, XNC can be replaced with linear network coding and packet ranking can be done. Also,

- To increase the efficiency and throughput, creative methods can be used and the results can be compared to this research.
- To examine the delay, the media-access protocols can be changed and competition-based patterns can be used.
- The consumed power can be examined and solutions can be proposed to increase the power in battery- and GPS-based vehicles.
- The network overhead can be examined with regard to the proposed method in this article.

## Supporting information

S1 Rar All raw results and simulation outputs. (RAR)

## References

[1] Al-Sultan S, Al-Doori MM, Al-Bayatti AH, Zedan H (2014) A comprehensive survey on vehicular Ad Hoc network. Journal of Network and Computer Applications 37: 380–392.

[2] Sharef BT, Alsaqour RA, Ismail M (2014) Vehicular communication ad hoc routing protocols: A survey. Journal of Network and Computer Applications 40: 363–396.

[3] Kenney JB (2011) Dedicated short-range communications (DSRC) standards in the United States. Proceedings of the IEEE 99: 1162–1182.

[4] Bai F, Stancil DD, Krishnan H. Toward understanding characteristics of dedicated short range communications (DSRC) from a perspective of vehicular network engineers; 2010 ACM pp. 329–340.

[5] Cheng L, Henty BE, Stancil DD, Bai F, Mudalige P (2007) Mobile vehicle-to-vehicle narrow-band channel measurement and characterization of the 5.9 GHz dedicated short range communication (DSRC) frequency band. IEEE Journal on Selected Areas in Communications 25.

[6] Bradai A, Ahmed T. ReViV: Selective Rebroadcast Mechanism for Video Streaming over VANET; 2014 18–21 5 2014 pp. 1–6.

[7] Barrekatain B, Aizaini Maarof M, Ariza Quintana A, Ghaeini HR (2013) Performance Evaluation of Routing Protocols in Live Video Streaming over Wireless Mesh Networks. jurnalteknologi (Scopus) 62: 101.

[8] Ghaeini HR, Akbari B, Barekatain B, Trivino‐Cabrera A (2015) Adaptive video protection in large scale peer‐to‐peer video streaming over mobile wireless mesh networks. International Journal of Communication Systems.

[9] Yen Y-S, Chao H-C, Chang R-S, Vasilakos A (2011) Flooding-limited and multi-constrained QoS multicast routing based on the genetic algorithm for MANETs. Mathematical and Computer Modelling 53: 2238–2250.

[10] Liang Z, Han-Chieh C, Vasilakos AV (2011) Joint Forensics-Scheduling Strategy for Delay-Sensitive Multimedia Applications over Heterogeneous Networks. Selected Areas in Communications, IEEE Journal on 29: 1358–1367.

[11] Vasilakos A, Ricudis C, Anagnostakis K, Pedryca W, Pitsillides A. Evolutionary-fuzzy prediction for strategic QoS routing in broadband networks; 1998 4–9 5 1998 pp. 1488–1493 vol.1482.

[12] Mazataud C, Bing B. A Practical Survey of H.264 Capabilities; 2009 11–13 5 2009 pp. 25–32.

[13] Ozbek N, Tunali T (2005) A survey on the H. 264/AVC standard. Turk J Elec Engin 13: 1–16.

[14] Tew Y, Wong K (2014) An Overview of Information Hiding in H.264/AVC Compressed Video. IEEE Transactions on Circuits and Systems for Video Technology 24: 305–319.

[15] Sowmyayani S, Rani J, Arockia P (2014) ADAPTIVE GOP STRUCTURE TO H. 264/AVC BASED ON SCENE CHANGE. ICTACT Journal on Image & Video Processing 5.

[16] Barekatain B, Maarof MA, Quintana AA, Cabrera AT (2013) GREENIE: a novel hybrid routing protocol for efficient video streaming over wireless mesh networks. EURASIP Journal on Wireless Communications and Networking 2013: 1.

[17] Barekatain B, bin Maarof MA. Network coding efficiency in live video streaming over Peer-to-Peer Mesh networks; 2011 IEEE pp. 1–7.

[18] Barekatain B, Khezrimotlagh D, Aizaini Maarof M, Ghaeini HR, Salleh S, et al (2013) MATIN: A Random Network Coding Based Framework for High Quality Peer-to-Peer Live Video Streaming. PLoS ONE 8: e69844 10.1371/journal.pone.0069844 23940530 PMC3734139

[19] Ramzan N, Park H, Izquierdo E (2012) Video streaming over P2P networks: Challenges and opportunities. Signal Processing: Image Communication 27: 401–411.

[20] Malatras A (2015) State-of-the-art survey on P2P overlay networks in pervasive computing environments. Journal of Network and Computer Applications 55: 1–23.

[21] Zong N (2008) Survey and Problem Statement of P2P Streaming. IETF PPSP bof.

[22] Pandey T, Garg D, Gore MM (2014) Structured P2P Overlay of Mobile Brokers for Realizing Publish/Subscribe Communication in VANET. The Scientific World Journal 2014. 10.1155/2014/136365 PMC3913288 24523629

[23] Barekatain B, Khezrimotlagh D, Maarof MA, Ghaeini HR, Quintana AA, et al (2015) Efficient P2P live video streaming over hybrid WMNs using random network coding. Wireless Personal Communications 80: 1761–1789.

[24] Ghaeini HR, Akbari B, Barekatain B (2013) An adaptive packet loss recovery method for peer-to-peer video streaming over wireless mesh network Emerging Technologies for Information Systems, Computing, and Management: Springer pp. 713–721.

[25] Ahlswede R, Ning C, Li SYR, Yeung RW (2000) Network information flow. Information Theory, IEEE Transactions on 46: 1204–1216.

[26] Ahmed SA, Ariffin SH, Fisal N (2015) Network coding techniques for VANET advertising applications. EURASIP Journal on Wireless Communications and Networking 2015: 1–13.

[27] Zhenni F, Yanmin Z, Qian Z, Min G. Exploiting Network Coding for Data Availability in Vehicular Networks: Issues and Opportunities; 2012 14–16 12 2012 pp. 24–30.

[28] Nguyen K, Nguyen T, Cheung S-C (2010) Video streaming with network coding. Journal of Signal Processing Systems 59: 319–333.

[29] Fiandrotti A, Zezza S, Magli E Complexity-Adaptive Random Network Coding for Peer-to-Peer Video Streaming.

[30] Pedersen HA, Sassatelli L, Aparicio-Pardo R. Random linear coded distributed caching for video streaming over D2D; 2018 IEEE pp. 1–8.

[31] Huang S, Izquierdo E, Hao P (2017) Adaptive packet scheduling for scalable video streaming with network coding. Journal of Visual Communication and Image Representation 43: 10–20.

[32] Drago M, Azzino T, Polese M, Stefanović Č, Zorzi M. Reliable Video Streaming over mmWave with Multi Connectivity and Network Coding; 2018 IEEE pp. 508–512.

[33] Katti S, Rahul H, Hu W, Katabi D, Médard M, et al (2008) XORs in the air: practical wireless network coding. IEEE/ACM Transactions on Networking (ToN) 16: 497–510.

[34] Prashanthi V, Babu DS, Rao CG (2016) Survey on Network Coding-aware Routing. International Journal 5.

[35] Bassoli R, Marques H, Rodriguez J, Shum KW, Tafazolli R (2013) Network Coding Theory: A Survey. IEEE Communications Surveys & Tutorials 15: 1950–1978.

[36] Rhaiem OB, Chaari L (2017) Information transmission based on network coding over wireless networks: a survey. Telecommunication Systems 65: 551–565.

[37] Jamil F, Javaid A, Umer T, Rehmani MH (2017) A comprehensive survey of network coding in vehicular ad-hoc networks. Wireless Networks 23: 2395–2414.

[38] Naeem A, Rehmani MH, Saleem Y, Rashid I, Crespi N (2017) Network coding in cognitive radio networks: A comprehensive survey. IEEE Commun Surveys Tuts 19: 1945–1973.

[39] Winger JL (2015) A Survey of Network Coding and Applications.

[40] Macharis C, Springael J, De Brucker K, Verbeke A (2004) PROMETHEE and AHP: The design of operational synergies in multicriteria analysis.: Strengthening PROMETHEE with ideas of AHP. European Journal of Operational Research 153: 307–317.

[41] Tavana M, Hatami-Marbini A (2011) A group AHP-TOPSIS framework for human spaceflight mission planning at NASA. Expert Systems with Applications 38: 13588–13603.

[42] Astudillo D, Chaput E, Beylot AL. Pseudo Random Network Coding in Infrastructure to Vehicle Environment for Data Download; 2015 14–16 7 2015 pp. 331–336.

[43] Qing W, Pingyi F, Letaief KB (2012) On the Joint V2I and V2V Scheduling for Cooperative VANETs With Network Coding. Vehicular Technology, IEEE Transactions on 61: 62–73.

[44] Firooz MH, Roy S. Collaborative downloading in VANET using Network Coding; 2012 10–15 6 2012 pp. 4584–4588.

[45] Hong J-L, Wang T-P, Hsieh Y-J (2014) Opportunistic Delayed Transmission for Network Coding in Vehicular Wireless Networks. Proceedings of the Asia-Pacific Advanced Network 38: 34–41.

[46] Sepulcre M, Gozalvez J, Gisbert JR. On the potential of network coding for cooperative awareness in vehicular networks; 2015 14–16 12 2015 pp. 143–148.

[47] Sahu PK, Hafid A, Cherkaoui S. Congestion control in vehicular networks using network coding; 2014 10–14 6 2014 pp. 2736–2741.

[48] Sahu PK, Hafid A, Cherkaoui S. Inter street interference cancelation in urban vehicular networks using network coding; 2014 8–12 12 2014 pp. 374–379.

[49] Khan S, Alam M, N M, llner Fr M, et al Cooperation and network coding based MAC protocol for VANETs; 2015 16–18 12 2015 pp. 64–67.

[50] Saaty TL (1990) How to make a decision: the analytic hierarchy process. European journal of operational research 48: 9–26. 10.1016/0377-2217(90)90060-o 11659401

[51] Bogdanovic D, Nikolic D, Ilic I (2012) Mining method selection by integrated AHP and PROMETHEE method. Anais da Academia Brasileira de Ciencias 84: 219–233. 22441612 10.1590/s0001-37652012000100023

[52] Shih H-S, Shyur H-J, Lee ES (2007) An extension of TOPSIS for group decision making. Mathematical and Computer Modelling 45: 801–813.

[53] Deng H, Yeh C-H, Willis RJ (2000) Inter-company comparison using modified TOPSIS with objective weights. Computers & Operations Research 27: 963–973.

[54] Olson DL (2004) Comparison of weights in TOPSIS models. Mathematical and Computer Modelling 40: 721–727.

[55] Chowdhury NM (2016) NETCODE: an XOR-based warning dissemination scheme for vehicular wireless networks: University of Glasgow.

[56] Achour I, Bejaoui T, Tabbane S. Network coding approach for vehicle-to-vehicle communication: Principles, protocols and benefits; 2014 17–19 9 2014 pp. 154–159.

[57] Mousavi SM, Rabiee HR, Moshref M, Dabirmoghaddam A. Mobisim: A framework for simulation of mobility models in mobile ad-hoc networks; 2007 IEEE pp. 82–82.

